# Application of machine learning for personalized advice about workers’ lifestyle changes to improve sustainable employability: Development and evaluation of a decision support tool

**DOI:** 10.1177/10519815261490878

**Published:** 2026-10-13

**Authors:** Marianne WMC. Six Dijkstra, Remko Soer, Etto L. Salomons, André H.J. Bieleman, Michiel F. Reneman, Douglas P. Gross

**Affiliations:** 1 Department of Rehabilitation Medicine, 3647 University of Groningen, University Medical Center Groningen, Groningen, The Netherlands; 2 Department Health and Care, Research Group Smart Health, 2975 Saxion University of Applied Sciences, Enschede, The Netherlands; 3 Department of Anesthesiology, Groningen Pain Center, 3647 University of Groningen, University Medical Center Groningen, Groningen, The Netherlands; 4 School of Ambient Intelligence, 2975 Saxion University of Applied Sciences, Enschede, The Netherlands; 5 Department of Physical Therapy, 3158 University of Alberta, Edmonton, Canada

**Keywords:** occupational health, wellness and prevention, artificial intelligence, health promotion, decision support, life style, workplace, healthy aging

## Abstract

**Background:**

Occupational Health Check-ups (OHCs) have evolved from identifying work-related health risks to supporting sustainable employability through individualized lifestyle advice, but translating multidimensional demographic, occupational, and health characteristics into effective, personalized recommendations remains complex and lacks validated decision-support methods.

**Objective:**

Aims of this study are to: 1) develop a Machine Learning Decision Support Tool (ML-DST) based on workers’ demographic, occupational, and health characteristics to support personalized advice about lifestyle changes to improve sustainable employability, and 2) evaluate performance of the ML-DST.

**Methods:**

Real-world OHC data from a population-based cohort (2010–2020) were used to develop ML-DST models providing lifestyle advice (physical activity, smoking, alcohol, nutrition, relaxation, or no change) to improve workability and vitality. Paired baseline and 1–4-year follow-up data were used to train and evaluate 12 models. Per model, three rule-based algorithms were applied. Performance was considered useful when both True Positive Rate (TPR) and macroF1 score were ≥0.7. The best performing algorithm is proposed per model for inclusion in the final ML-DST.

**Results:**

Of 20,440 individual OHCs, 3412 paired sets were included. Best TPRs ranged from 0.51 to 0.71. One model achieved TPR and macroF1 scores ≥0.7, supporting nutritional behavior change to improve vitality; however, its profiles were not conceptually meaningful. The other 11 models did not achieve useful quantitative performance.

**Conclusion:**

Training algorithms for an ML-DST to support advice about lifestyle changes to improve long-term sustainable employability using data from regular Occupational Health Check-ups did not lead to useful model performance for improving workability and vitality.

## Introduction

In an aging workforce, improving workers’ ability to stay at work, while preserving health, wellbeing and employability is a public health challenge.^1–3^ Globally, workers are staying at work longer, however employability of aging workers is influenced by the natural aging process, lifestyle choices and chronic disease.^4–6^ Poor health is the most prevalent reason for exiting work prematurely.^7^ Unhealthy lifestyle behaviors are related to sickness absence^8^ and increase risk of chronic disease,^9^ supporting the use of a broad assessment to inform provision of advice regarding sustainable employability (SE).

A regularly used method to support workers with regard to improving or sustaining health and employability, is an individual Occupational Health Check-up (OHC), followed by provision of advice aimed at improving health through changes in work characteristics or lifestyle.^10^ Lifestyle factors such as obesity, smoking and physical leisure time inactivity are often prevalent in combination with work related risk factors^11^ and combining these might lead to better risk assessment and subsequent advice to improve SE. In the Netherlands, employers are legally obligated to offer OHCs, but participation is voluntary for employees.

An OHC is often administered using questionnaires on lifestyle, work, and personal characteristics combined with biometric health measures. Despite the use of valid measures, supportive legislation, and well-trained professional consultants performing the OHCs, their effectiveness for enhancing SE has not previously been demonstrated.^12^ Several reasons could explain why OHCs have not demonstrated effectiveness. First, validation is typically performed at the level of individual instruments rather than the overall OHC as implemented in real-world practice. This is relevant because the predictive value of combinations of measures for SE are unknown. Moreover, it is unclear whether lifestyle and work-related risks together lead to optimal individual advice.^11^ Second, not all measures are validated for SE, but for other work-related outcomes (i.e., Body Mass Index or engagement). It is possible that some measures are redundant when screening for SE. Last, interpretation of OHC results regarding SE can be complex and occasionally discrepant, for example when workability risks are identified in the absence of work stress or strain risks. Combining multidimensional constructs to inform individual advice, aimed at improving long-term SE is complex and there are currently no standardized decision protocols available. This can lead to large interindividual differences between Occupational Health Professionals’ (OHP) recommendations in practice. Observed variation can lead to differences in effectiveness of OHCs for SE and potential inequality between workers.^13^

Hence, there is a need to support OHPs to provide individualized, objective advice for maintaining or improving workers’ employability. One promising approach is through development of algorithms that process diverse data and recognize complex multidimensional non-linear patterns in health settings.^14,15^ Validated Machine Learning-based Decision Support Tools (ML-DSTs) for SE are not available for the occupational health setting. Knowledge about the performance of ML-DSTs is essential for proper interpretation of results when used to inform advice to increase worker's SE by OHPs.^16^

The aims of this study were to: 1) develop an ML-DST based on worker demographic, occupational, and health characteristics to support provision of personalized advice about lifestyle changes to improve sustainable employability, and 2) evaluate performance of the ML-DST.

## Methods

The process of applying supervised ML to a real-world problem described by Kotsiantis^17^ was applied to this study (Figure 1). Data collection and ML processes are described below, while interconnections between both are represented in Figure 1. This study was performed in accordance with the 1964 Declaration of Helsinki and its later amendments. The Medical Ethical Committee of the University Medical Centre Groningen in the Netherlands provided a waiver as all data were gathered within usual care, participation was voluntary, and only anonymized cases were analyzed. The study is reported according to the STROBE Statement for observational cohort studies.^18^

**Figure 1.**
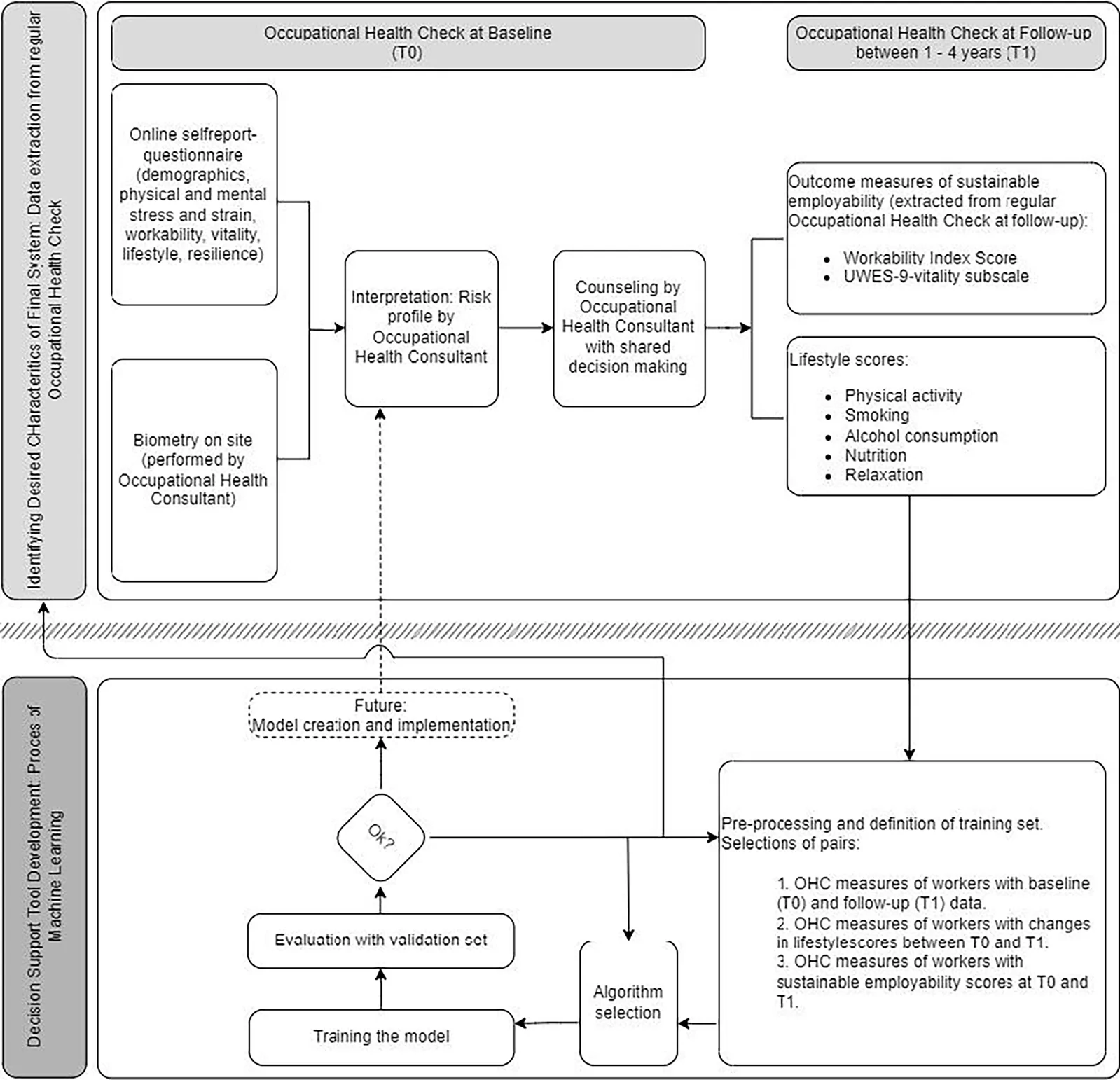
*Above:* The process of collecting identified data at baseline (T0) and follow-up (T1) from the regular occupational health check*. Below*: The process of machine learning (according to Kotsiantis (13)). The implementation of the ML-DST is beyond the scope of this study and presented with small dotted lines. *DST = Decision Support Tool; UWES-9* *=* *Utrecht Work Engagement Scale (9 item version); OHC = Occupational Health Check.*

### Section 1: Identification and collection of data

#### Study design and setting:

A population-based cohort study was conducted, with prospective and historical data collection. We extracted data from an occupational health supplier's (OHSs) database (proprietary, Informens B.V.) of real-world OHCs. The OHSs services 18 moderate and large companies in a variety of sectors (education, transport, automotive industry, housing association, water authority, food processing, waste processing, metal industry, retail, finance) in the Netherlands. The project was initiated in November 2015 and the cohort was followed until July 2020. Historical workers’ data starting October 2010 were included to expand the database. Thus, the entire data collection period was October 2010 to July 2020.

In the Netherlands, employers are required to offer their employees an OHC *at least* every four years. The follow-up period in our study was between one and four years, depending on preference of companies affiliated with the participating OHS. Participation was voluntary for workers. As part of the OHC workers completed a questionnaire at home and a biometric health assessment by the OHP. Results were entered into the OHS's database. The full procedure is presented in Figure 1. For development of the ML-DST, all raw data were anonymized by the OHS, compiled into an excel file, and shared with the researchers.

#### Participants undergoing workers’ health assessment:

All workers 18 years and older were invited to undergo OHC (in the Netherlands retirement age was 65 years until 2018 and has gradually shifted to 66 years and 4 months in 2020).

#### Measures

All data were extracted from the OHS's database:

*1) Predictors:* 35 OHC baseline measures were selected based on theory or expert opinion for their potential influence on long term effects on workers SE or health^19^ and described elsewhere.^20^ This included personal characteristics, occupational characteristics, work-related physical and mental stress and strain factors, individual work and health-related factors, and lifestyle (see Table 1).
*2) Lifestyle:* For changes in lifestyle over time, the results of questionnaires between baseline and follow-up measures were compared. The lifestyle questionnaires asked about Physical Activity, Smoking, Alcohol, Nutrition, and Recreation. They are descriptive in nature and reflect national guidelines^21^ based on general evidence-based knowledge about associations between lifestyle and health (see Appendix A). Scores were categorized using an ordinal scale as high risk, moderate risk, or low risk for decreased health.
*3) Outcome measures*: SE was defined as self-reported workability and vitality.^22^ Workability was measured with the WorkAbility Index (WAI) as an ordinal scale between 7-49.^23^ The WAI score was categorized as poor (7-27), moderate (28-36), good (37-43) or excellent (44-49).^24^ The vitality subscale of the 9-item-Utrecht Work Engagement Scale (UWES-9-vitality) was used as a measure of vitality. The UWES-9-vitality score is categorized as very low (≤2), low (2.01-3.25), moderate (3.26-4.80), high (4.81-5.65) and very high (≥5.66).^25,26^

**Table 1.** Baseline descriptive characteristics of workers undergoing Occupational Health Check-ups (n = 3412).

Variable	Measure	Descriptive statistics
**Personal characteristics**		
Age (n = 3412)	Years	Mean 43.58 (sd 10.2)
Gender (n = 3412)	Male – Female	83.1% - 16.9%
Education level (n = 3385)	Very low – Low - Intermediate – High	3.3% - 16.4% - 43.7% - 36.4%
**Work characteristics**		
Job category WAI (n = 3130)	Workability Index – question main type of work: Mainly mental - Mental and physical – Mainly physical	59.9% – 24.6% – 15.5%
Irregular shifts (n = 3385)	Irregular – Regular	28,1% - 71.9%
**Individual work-related characteristics**		
Contract (n = 3384)	Fixed – Temporary	93.1% - 6.9%
Working hours/ week (n = 3379)	Hours/week	Median 40 (range 4-60)
Years employed (n = 3385)	Years	Median 7 (range 0-50)
**Sustainable employability**		
Workability (n = 3195)	Workability Index (scale 7 - 49)	Median 43 (range 19–49)
Vitality (n = 1713)	Utrecht Work Engagement Scale 9-vitality (scale 1–6)	Median 4.3 (range 0-6)
**Health characteristics**		
Cardiovascular risk-1 (n = 3091)	Blood Pressure Diastolic (mmHg)	Mean 82 (sd 10.3)
Cardiovascular risk-2 (n = 3093)	Blood Pressure Systolic (mmHg)	Mean 136 (sd 15.8)
Cardiovascular risk-3 (n-3053)	Blood Cholesterol (finger) (mmol/l)	Mean 5.1 (sd 1.1)
Diabetic risk (n = 3051)	Blood Glucose (finger) (mmol/l)	Median 5 (range 1.3-19.7)
Overweight-1 (n = 3095)	Body Mass Index (kg/m^2^)	Median 25.8 (range 17-46)
Overweight-2 (n = 3012)	Waist circumference (cm)	Mean 94 (sd 11)

Variable	Measure	High risk (%)	Moderate risk (%)	Not at risk (%)
Autonomic Nervous System functioning (n = 1359)	Heart Rate Variability; 1-min paced breathing (MHRR)	3.3	5.9	90.8
Psychological health (n = 1486)	General Health Questionnaire - 12	9,7	n.a.	37.2
Engagement (n = 1709)	Utrecht Work Engagement Scale – 9	0.9	7.7	91.4
**Work-related stress and strain**	Questionnaire on the Experience and Evaluation of Work(QEEW-scale 1-100)			
Need for Recovery n = 2914)	QEEW – Need for Recovery	17.7	14.0	68.3
Work pace (n = 2873)	QEEW – Workpace	4.4	14.5	81.0
Mental Strain (n = 3168)	QEEW- Mental strain	10.4	87.0	2.6
Physical Strain (n = 3168)	QEEW- Physical strain	8.3	44.9	46.8
**Lifestyle**				
Physical Activity (n = 3381)	Lifestyle questionnaire (proprietary)	47.9	9.1	43.0
Smoking (n = 3311)	Lifestyle questionnaire (proprietary)	15.8	1.9	82.3
Alcohol (n = 3310)	Lifestyle questionnaire (proprietary)	1.9	10.5	87.7
Nutrition (n = 3305)	Lifestyle questionnaire (proprietary)	14.9	38.6	46.5
Recreation (n = 3262)	Lifestyle questionnaire (proprietary)	36.3	14.2	49.5
**Occupational Health characteristics**				
Strength (n = 2057)	Pinch strength test	20.3	18.5	61.2
Flexibility Back (n = 2956)	Sit & Reach test	24.8	69.4	5.8
Physical endurance (n = 2388)	Harvard Steptest	36.1	17.0	46.9

*MHRR = Mean Heart Rate Range; QEEW = Questionnaire on the Experience and Evaluation of Work*; Lifestyle questionnaire is based on Guidelines of the National Institute for Public Health and the Environment- *Ministry of Health, Welfare and Sport* in the Netherlands.

#### Data handling

Only workers who participated in two OHC's with a follow-up period between one and four years were included. Missing predictors were allowed because this represents the most likely real-life situation after implementation of the ML-DST. Cases with scores on at least one outcome measure at follow-up were included. The algorithms were trained to predict improvement of SE, which was classified as a binary variable: ‘improved’ when the worker scored a higher category of workability/vitality at follow-up and ‘not improved’ when the worker scored in the same category or lower at follow-up. Lifestyle was similarly classified as ‘improved’ or ‘not improved’.

#### Descriptive statistics

Descriptive analyses were calculated with SPSSv26. This included means and standard deviations for normally distributed continuous variables, medians and range for skewed continuous variables, and percentages for nominal and ordinal data.

### Section 2: ML-DST development, training, testing

#### Data pre-processing and definition of the training set

For ML-training purposes, we categorized data based on three criteria: 1) workers had an assessment at baseline *and* follow-up, 2) when criterion one was met, workers were categorized into groups based on lifestyle changes and 3) when the first two criteria were met, only workers with scores for workability and/or vitality at both baseline and follow-up were selected (Figure 2, respectively pre-processing step 1, 2 and 3). Some workers improved on multiple lifestyle factors and were therefore categorized into multiple subgroups. This led to 12 data-subsets with six lifestyle factors (change in physical activity, smoking, alcohol consumption, nutrition, relaxation, or no lifestyle change) for two outcomes of SE (workability and vitality).

**Figure 2.**
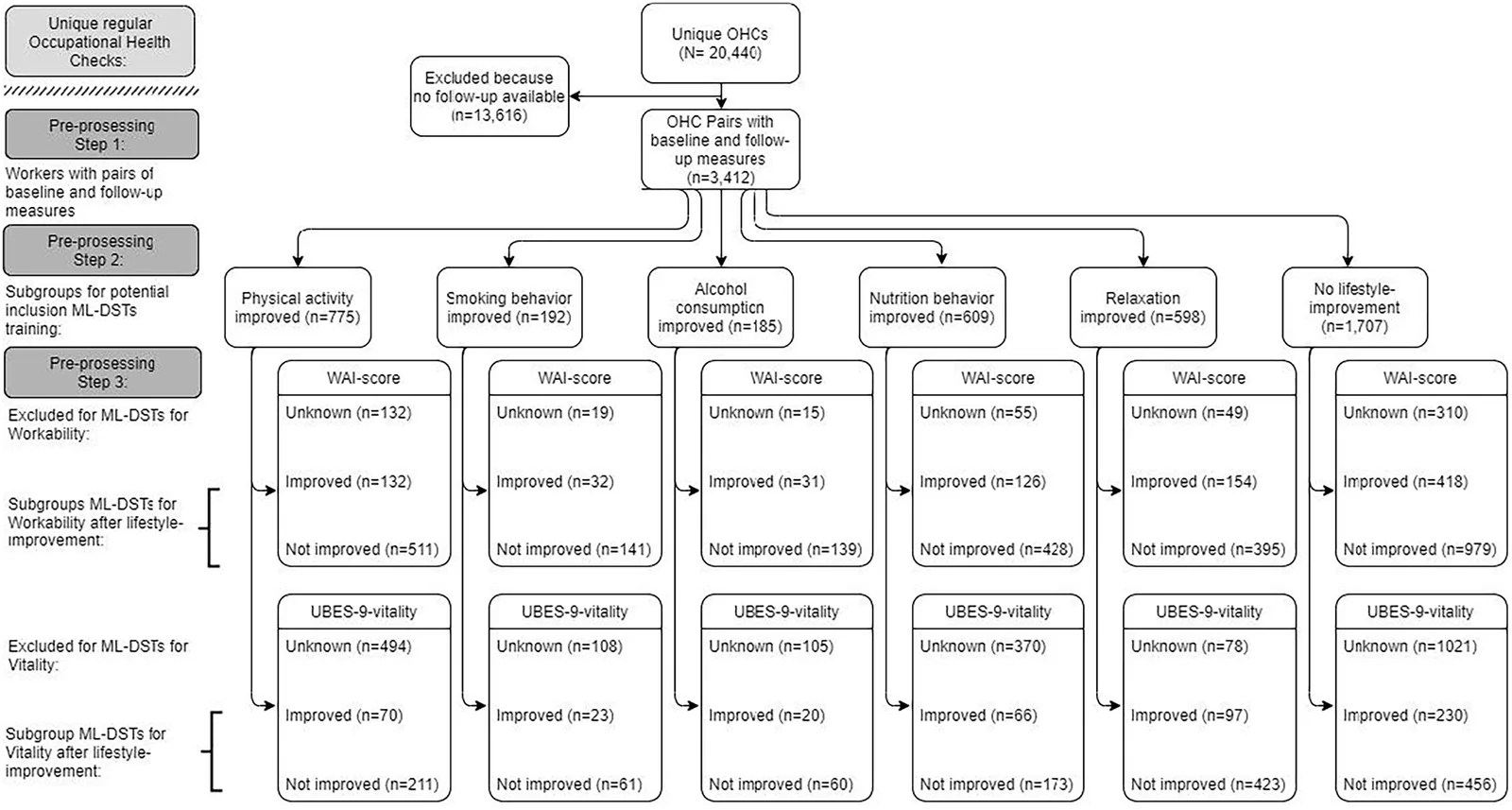
Flowchart of workers completing a workers’ health assessment for the ml-algorithms. Firstly, workers with follow up data were included (OHC Pairs). Secondly, subgroups with improved lifestyle were allocated for potential inclusion for the ML development. Thirdly the final subgroups were selected for ML-DST training sets for workability and vitality for all measures of lifestyle.

#### Algorithm selection

We developed one model for each data-subset, resulting in 12 models (Figure 2). For each model, two classes were possible: ‘improved’ SE or ‘not improved’ SE. Common ML techniques (SMOTE) were applied to overcome imbalance between classes.^27,28^

For each model, we trained three supervised learning algorithms and selected the best performing (classifying). For clinically relevant interpretation of the suggested recommendations, the specific advice should be transparent. Therefore, only rule-based and explainable algorithms were used: Ripper (Repeated Incremental Pruning to Produce Error Reduction), Ridor (Ripple Down Rule Learner) and C4.5 (creates a decision tree). Machine learning analyses were conducted using the Waikato Environment for Knowledge Analysis (WEKA).^29^

#### Training the models for the ML-DST

A cross-validation technique was used to develop the models.^16^ All 12 subsets of data were partitioned into three random cross-validation sets with equal distributions. Each algorithm was trained on two cross-validation sets and validated on the third set. This was repeated for three combinations, where each cross-validation was used once as validation-set. Per algorithm, the mean of three test-runs is calculated for the performance measures that are described in the next paragraph.

#### Evaluation of the ML-DST model performances

Predicted classifier outcomes (based on the training sets) were compared with true results in the validation set in a 2 × 2 confusion matrix. The matrix was created for all 12 subsets; Appendix B shows an example for workability. Three evaluation techniques were used: *True Positive Rate, True Negative Rate* and *macroF_1_score (*described below).^30^ Appendix B shows the calculations.

*1) True Positive Rate* (*TPR*), shows the fraction of correct predicted cases in the class ‘improved’. In other words, the percentage of workers who benefited from a lifestyle change and showed real, observed improvements in workability or vitality that was correctly predicted by the classifier.
*2) True Negative Rate* (*TNR*), shows the fraction of correct predicted cases in the class ‘not-improved’. In other words, the percentage of workers with a lifestyle change who did not show real, observed improvement in workability or vitality, which was also predicted by the classifier.

Values of TPR and TNR are between 0 and 1; closer to 1 is better. Ideally, the classifier would perfectly predict who would benefit from a lifestyle change to increase their SE (TPR=1) and who would *not* benefit from a lifestyle change (TNR=1). A value of 0.5 indicates that the predictor performs randomly.^30^ Generally, a TPR close to 1 is not found when prevalence is low, as can be expected for decreased SE in this relatively healthy population.^30,31^ Therefore, we considered a value of 0.7 or higher as good.

*3)* MacroF_1_score is the balanced measure that evaluates the relations between the observed classes (improved or not-improved) of the validation-sets and the classes predicted by an ML-DST classifier based on a per-class average (see Appendix B). The value is between 0 and 1; closer to 1 is better. A value ≥0.7 was considered potentially useful.^30^

To evaluate performance of the algorithms, the outcome measures were interpreted with respect to clinical implications. The consequence of a low TPR would be that a worker will not be advised to improve lifestyle, although an improvement in lifestyle would have improved the workers’ SE. Therefore, we consider TPR as the most important evaluative measure. When TPR score is ≥0.7, the next step is to evaluate the macroF_1_score, in which the classes (improved/not improved) have equal weight and TNR is also considered. When macroF_1_score is ≥0.7, predictive value of the algorithm was deemed potentially useful.

## Results

### Section 1: Collection of required data

#### Participants undergoing occupational health check-up

Of 20,440 unique OHCs in the database, 3412 workers participated in both baseline and follow-up assessments. This sample of 3412 workers were included in subsequent analyses (OHC-Pairs, pre-processing step 1, Figure 2). Baseline descriptive characteristics of included workers are presented in Table 1. WAI was completed by 3221 workers at baseline, with 44% scoring excellent, 46% good, 9% moderate and 0.1% scoring poorly. The UWES-9-vitality at baseline was completed by 1713 workers, with 8% scoring very high, 20.1% high, 55% moderate, 13.4% low and 3% scoring very low.

At follow-up, WAI-score was improved for 16% of the workers, not improved for 64% and for 30% it was unknown because WAI score was unavailable for baseline and/or follow-up measurement. UWES-9-vitality was handled likewise; 8% showed improved vitality, 28% did not improve and 64% were missing at follow-up. A post hoc test revealed that although statistically significant differences were found in baseline WAI and UBES-9-vitality scores between the included and excluded workers, the differences were not clinically relevant (median WAI score 43 at both T0 and T1; mean UBES-8-vitality score 4.2 versus 4.1).

### Section 2: ML-DST development process

#### Definition of the training set for ML-DST

The flowchart in Figure 2 shows that 3412 workers were categorized by improved lifestyle factors. The largest group (n = 1,707, 50%) was categorized by ‘no lifestyle improvement’ on any lifestyle factor. Consequently, the other 50% changed at least one lifestyle factor. For smoking and alcohol consumption, approximately one third of workers were included (respectively n = 192 and n = 185) in relation to the groups for physical activity (n = 775), nutrition (n = 609) and relaxation (n = 598). For pre-processing step 3, the final subgroups for model training were defined for workability and vitality (improved or not improved) for each lifestyle factor. For example, figure 2 shows that 775 workers improved their physical activity lifestyle. One hundred thirty-two of these workers also improved on WAI score, while 511 did not.

#### Evaluation of model performance

Only two algorithms performed above TPR≥0.7 indicating potentially useful performance. These two algorithms predicted for whom a positive lifestyle change will increase *vitality* score: Alcohol consumption (TPR for vitality = 0.70 with C4.5), and Nutrition (TPR for vitality = 0,71 with C4.5). For workability, all algorithms performed low (TPR=0.41) to moderate (TPR = 0.65)

In contrast, all algorithms, except one, were able to identify which workers did not improve their workability or vitality when lifestyle improved (TNR ≥ 0.7). The only algorithm with TNR below cutoff was the C4.5 for vitality and Alcohol consumption (TNR = 0.67), which also had a good TPR score. See table 2 for the TPR, TNR and macroF_1_scores of the algorithms.

**Table 2.** Evaluation of algorithm performance for the 12 developed models.

Models		Evaluative measures									
Improved Lifestyle-factor	Measure of Sustainable Employability	True Positive Rate (Recall-class ‘improved’)			True Negative Rate (Recall-class ‘not improved’)			MacroF_1_score			Useful algorithm for model
		Ripper	Ridor	C4.5	Ripper	Ridor	C4.5	Ripper	Ridor	C4.5	
Physical activity	Workability	**0** **.** **53**	0.43	0.45	0.90*	0.98*	0.93*	0.73*	0.72*	0.70*	all rejected ‡
	Vitality	0.55	0.54	**0**.**65**	0.88*	0.87*	0.78*	0.72*	0.71*	0.72*	all rejected ‡
Smoking	Workability	0.44	0.41	**0**.**52**	0.90*	0.94*	0.91*	0.67	0.69	0.73*	all rejected ‡
	Vitality	0.61	**0**.**69**	0.59	0.85*	0.87*	0.84*	0.73*	0.79*	0.71*	all rejected ‡
Alcohol consumption	Workability	**0**.**65**	0.47	0.50	0.81*	0.94*	0.84*	0.73*	0.73*	0.68*	all rejected ‡
	Vitality	0.65	0.43	**0**.70*	0.78*	0.88*	0.67	0.71*	0.65	0.68	all rejected †
Nutrition	Workability	**0**.**58**	0.50	0.54	0.93*	0.96*	0.96*	0.77*	0.74*	0.77*	all rejected ‡
	Vitality	0.60	0.51	**0**.71*	0.79*	0.92*	0.79*	0.70*	0.71*	0.75*	C4.5
Relaxation	Workability	**0**.**58**	0.51	0.55	0.91*	0.96*	0.91*	0.75*	0.74*	0.73*	all rejected ‡
	Vitality	**0**.**69**	0.67	0.65	0.80*	0.85*	0.80*	0.75*	0.76*	0.73*	all rejected ‡
No lifestyle change	Workability	**0**.**51**	0.45	0.45	0.94*	0.97*	0.96*	0.75*	0.73*	0.73*	all rejected ‡
	Vitality	0.53	0.44	**0**.**54**	0.87*	0.90*	0.81*	0.71*	0.68	0.68	all rejected ‡

* = performance ≥ 0.7.

† = no algorithm selected because C4.5 - TPR ≥ 0.7, but C4.5 - macroF_1_score ≤ 0.7.

‡ = no algorithm selected because TPR ≤ 0.7.

Per model, results for algorithm with best performing TPR are printed bold.

The low TNR and borderline TPR of the Vitality-Alcohol consumption model resulted in a low macroF_1_score (0.68). For the final model selection, the criteria for useful performance was both a TPR and an overall performance (macroF1 score) ≥ 0.70. Only the model for Vitality-Nutrition with the C4.5 algorithm met the selection criteria (with TPR = 0.71 and macroF_1_score = 0.75) for inclusion in the ML-DST. For prediction for whom vitality will increase after a positive change in nutritional behavior, the trained C4.5 algorithm is useful, based on the performance criteria.

C4.5 constructs a decision tree (Appendix C), leading to detailed, multi-dimensional representations of workers based on various characteristics (profiles) of who will benefit and who will not. The model presents 28 profiles. E.g., the first profile in appendix C is followed: when vitality score is below 3.27 the next decision is based on gender. For a male with high-risk systolic blood-pressure, the advice is to change nutritional behavior to improve vitality. For a female, the advice is based on smoking behavior: with high risk smoking behavior the advice is not to improve nutritional behavior. Fifteen OHC measures are included in the decision tree, the other 20 measures did not contribute meaningfully.

## Discussion

Using a large database containing workers undergoing OHCs over a full decade, algorithms could not be trained to create a clinically useful ML-DST. The usefulness of only one model met our quantitative performance indicators. This algorithm supports decisions for which workers are likely to see improved vitality from nutritional behavior-change. For all other lifestyle changes to improve vitality or workability, performance of the models varied between low and moderate. These eleven models were not useful for the ML-DST and contribute little additional value beyond random guessing.

An important finding for future development of ML-DSTs in this context, was that despite useful quantitative performance of one model, this model did not provide conceptually *meaningful* profiles. For instance, a female with vitality score below moderate (<3.27) and a moderate health risk due to smoking is advised to change nutritional behavior, while the same female with high risk due to smoking is advised not to change nutritional behavior. There is no evidence supporting these or many other model profiles. The results underscore the importance of rule based algorithms that allow direct examination of decision logic, rather than black-box ML techniques such as deep learning, or neural networks. Although such techniques often demonstrate better quantitative performance, their black-box nature limits the ability to inspect the decision pathways leading to specific advice. Methods such as SHapley Additive exPlanations (SHAP) offer post-hoc explanations of model behavior but do not enable examination of the model's internal mechanisms.^32,33^

One potential explanation for our ‘negative’ results could be that information gathered as part of OHCs conducted in real-world settings do not contribute to future SE through changes in lifestyle. Alternatively, these results could be due to limitations in our data collection, pre-processing, or analytical approaches.^17^ We will discuss each of these to inform future developments.

The measures used in our OHCs may not be sufficient for predicting SE. Our measurement protocol was chosen from an occupational health perspective used in real world health services, A more comprehensive assessment approach may be better for improving SE or guiding lifestyle changes, such as job-fit or employability,^22,34^ good quality and accessible health services, income security and social protection, decent living conditions and social and human capital.^3^ Another category of measures that could be considered are cumulative exposures to strain, which are related to the lifespan perspective of SE.^34^ Cumulative exposures for a longer period in life can have different effects on disability pensions than short-term exposures,^35^ and may be more likely to influence SE.

Choosing lifestyle-changes as ‘interventions’ for the ML-DST was supported by evidence that lifestyle is related to many health risks upon exit from work.^8,9,36–38^ However, it is not easy to change lifestyle behavior and the effectiveness of lifestyle interventions at work have been modest.^36^ In their review of 74,296 participants in a comparable population, Virtanen et al.^8^ found that lifestyle-related factors were associated with sickness absence due to several specific diseases and are important for work capacity among the working-age population, but the nature of the associations was unclear. We attempted to add to this knowledge by predicting for whom a positive lifestyle-change would be beneficial to increase SE, justifying the choice of lifestyle-changes as input for the ML-DST. We trained the algorithms for separate lifestyle-changes and workers could be allocated to more sub-datasets. From a clinical perspective, it would have been valuable to train algorithms for combinations of lifestyle-changes.^39^ However, this would have led to even smaller sub-datasets, which is not feasible with the current database. Additionally, work related interventions would also be interesting to include if the ML-DST is developed further.^40^ Lifestyle-factors and work related-risks co-exist as risk factors for absence from work, but it is unclear how this may lead to valid advice for individual workers.^11^

An alternative explanation for our results could be the unanticipated high level high level of missing data, especially missing outcome data for vitality (64%). Although all partners and stakeholders voiced commitment to the project, collection of survey data is problematic when voluntary and done in the context of usual business. Studies in occupational health where baseline surveys are linked to registers of disability at follow-up, show higher levels of participation.^41,42^ Our focus on SE instead of disability did not allow us to use register data and was completely dependent on completion of surveys or questionnaires. As a consequence, the subgroups to train the algorithms after pre-processing the data, were small for ML purposes, although the sample of workers (n = 3412) who followed-up was substantial. There were more workers *without* increased SE than *with* increased SE as a natural result of observing relatively healthy workers, resulting in an unbalanced dataset that was more difficult to train. The technique that we used to overcome this imbalance (SMOTE), synthesizes more cases in the minority class based on existing minority instances,^27^ with a disadvantage that unrealistic cases might be created. Despite this disadvantage, SMOTE was preferred over another resampling method (Resample), which cuts down the majority classes, decreases the sample of real cases, and leads to fewer relevant cases included. A post hoc sensitivity analysis with Resample revealed that overall performance was slightly lower than with the use of SMOTE. Despite using SMOTE, the high TNR scores and the confusion matrices reflect that the ‘not improved’ class is still larger than the ‘improved’ class. A post hoc test of algorithms without balancers, revealed an overall performance only slightly better than 0.5. Training the models post-hoc with neural networks also did not increase performance. These post-hoc tests support the suggestion that more cases are required for a valid performance of the ML-DSTs.

### Strengths and limitations

This study included data from a large number of OHCs conducted within a real-world context, with a long follow-up period. To our knowledge, this is the first study using ML techniques to combine complex multidimensional data from actual workers using measures that are regularly used in real life to support workers to improve workability or vitality. Real-world data were used to increase content validity of the ML-DST when implemented by the OHS, yet based on our study results, no meaningful profiles were found to support advice for lifestyle changes to increase workability or vitality.

There are general weaknesses of observational research, such as a potential for selection bias. The voluntary nature of the assessment for both workers and companies possibly caused a selection and may not be generalizable. Our working population was inherently healthy and workers on sickleave were unlikely to participate, causing sampling bias through a healthy-worker effect. Previous studies have demonstrated that poor health,^1,38,43,44^ sickness absence, and presenteeism^45^ were associated with *un*sustainable employability. Missing workers at risk could have contributed to the high TNR values and relevant rules that lead to improved SE may not have been detected. Furthermore, using questionnaires could have led to under- or overestimated results through social desirability causing information bias. It could be interesting to explore technological systems to monitor workers’ lifestyle and use these data for the ML-DST development in future studies. Another serious difficulty that came forward during this study is the collection of data by numerous stakeholders in the constantly changing occupational context, which might limit the applicability of data mining procedures.^46^

Performance of the algorithms might have been affected by the ceiling effect of workability and vitality scores and by category cutoffs.^34,47^ The four WAI-categories are large and changing to another category might be difficult to achieve in the follow-up of 1 or 2 years, which was previously found for physical activity interventions,^48^ especially for workers with good workability.^47^ When two workers start with good WAI and improve 6 points, they can be categorized as ‘not improved’ or ‘improved’ depending on the baseline measure (WAI=37 versus WAI=38). To our knowledge, the use of minimal detectable changes for individual changes in WAI score have not been validated yet. It would be interesting to reconsider the cut-off scores and use minimal detectable changes instead for the ML-DST. The same would apply for UWES-9-vitality scores, although more (five) categories were already used, allowing easier transfer to another category than for the WAI-scores. This might have led to the slightly better performance of the vitality models over the WAI models, although there were less cases with vitality scores.

### Concluding remarks

Training algorithms to support decisions about lifestyle changes to improve long-term SE using occupational, health, and personal characteristics did not lead to useful model performance for improving workability and vitality. The development of an ML-DST is an iterative process and suggestions for changes in the input dataset are provided. Use of other algorithms that would not require sub-datasets could increase performance, but have drawbacks related to transparency of the models.

## Data Availability

Meta data are available from open access studies. The raw and cleaned data supporting the findings of this study are available from the corresponding author upon reasonable request (Data Use Agreement). Researchers have custodianship over these data, while the Occupational Health Supplier retains ownership. All data are stored in the controlled access data storage system ‘Saxion Institutional Repository’ at Saxion University of Applied Sciences.

## Appendix A. Cut-off values of the lifestyle questionnaires

Cut-off values of the lifestyle questionnaires, which are described in the methods section of the text.

**Table:**

Cut off points	Not at risk (0 points)	Moderate risk (1 point)	High risk (2 points)
Activity	at least 5 days per week for 30 minutes low intensity activity AND at least 2 days per week 20 minutes intensive active	at least 5 days a week 30 minutes low intensity activity OR at least 2 days per week 20 minutes. intensive active	less than 5 days per week 30 minutes low intensity activity AND less than 2 days per week 20 minutes intensive active
Smoking	Non-smoker	≤2 days per week in combinations with ≤ 10 cigarettes per day	≥ 3 days per week or ≤2 days per week in combinations with ≥ 10 cigarettes per day
Alcohol	≤ 5 days per week and ≤ 15 drinks per week	> 5 days per week and ≤ 15 drinks per week OR ≤ 5 days per week > 15 drinks per week	> 5 days per week and >15 drinks per week
Nutrition	Nutrition behavior without undesirable attitude as mentioned at moderate and high risk	≤ 4 days per week 3 meals or ≤ 4 days per week 200 grams vegetables	≤ 1 day, week 3 meals and/or ≤ 1 day, week 200 grams vegetables and/or ≤ 4 days, week 3 meals plus ≤ 4 days, week 200 grams vegetables
Relaxation	Risk score is proprietary for OHS		

## Appendix B

Calculation of evaluative measures is based on the confusion matrix (Figure Appendix B).

In the confusion matrix, the number of cases with observed change in workability (columns) versus classified change in workability (rows) are presented. This enables calculation of evaluative measures for the ML-DST. The True Positive Rate and True Negative Rate are evaluative measures. Precision, TPR and TNR are used to calculate the evaluative measure *macroF_1_score* (Appendix B). TP = number of True Positive cases, FN = number of False Negative cases, FP = number of False Positive cases, TN = number of True Negative cases.

*1) True Positive Rate* (*TPR*), also known as *recall* for class ‘improved’, or ‘sensitivity’, shows the fraction of correct predicted cases in the class ‘improved’. In other words, the percentage of workers who benefited from a lifestyle change through real, observed improvements in workability or vitality which was correctly predicted by the classifier.

*2) True Negative Rate* (*TNR*), also known as *recall* for class “not improved”, shows the fraction of correct predicted cases in the class ‘not-improved’. In other words, the percentage of workers with a lifestyle change, who did not show real, observed improvement in workability or vitality, which was also predicted by the classifier.

Values of TPR and TNR are between 0 and 1; closer to 1 is better. Ideally, the classifier would perfectly predict who would benefit from a lifestyle change to increase their SE (TPR=1), and also who would *not* benefit from a lifestyle change to increase their SE (TNR=1). A value of 0.5 indicates that the predictor performs random guess (26). Generally, a TPR close to 1 is not found when prevalence is low, as can be expected for decreased SE in this relatively healthy population(26, 27). Therefore a value of 0.7 or higher is considered good in the current study.

*3) MacroF_1_score* is the balanced measure that evaluates the relations between the observed classes (improved or not-improved) of the validation-sets and the classes predicted by an ML-DST classifier based on a per-class average. It is the harmonic mean between precision and recall, where the average is calculated per-class and then averaged over both classes. The value is between 0 and 1; closer to 1 is better. A value of 0.7 or higher is considered useful(26).

Calculations for class ‘improved’:

$$Recall_{improved}=\frac{TP}{TP+FN}$$

$$Precision_{improved}=\frac{\mathrm{TP}}{TP+FP}$$

$$F1_{improved}=2*\frac{Precision_{improved}*Recall_{improved}}{Precision_{improved}+Recall_{improved}}=\frac{\mathrm{TP}}{TP+\frac{1}{2}(FP+FN)}$$

Calculations for class ‘not improved’:

$$Recall_{not\;impoved}=\frac{\mathrm{TN}}{TN+FP}$$

$$Precision_{not\;improved}=\frac{TN}{TN+FN}$$

$$F1_{not\;improved}=2*\frac{Precision_{not\;improved}*Recall_{not\;improved}}{Precision_{not\;improved}+Recall_{not\;improved}}=\frac{\mathrm{TN}}{TN+\frac{1}{2}(FP+FN)}$$

Calculation for overall performance:

$$Macro\;F1\;score=\frac{1}{2}(F1_{improved}+F1_{not\;improved})$$

## Appendix C. Decision tree for Vitality – Nutritional Behavior

For the final Decision Support Tool the C4.5 algorithm was selected for the final model to predict who will benefit from an change in nutritional behavior to change vitality in future. C4.5 constructs a decision tree. The decision tree is presented as a set of rules. (number of cases in test-set/number of wrong classified cases).

The rules in the selected algorithm for the final model of the ML-DST are described below.

R = red = high risk; O = orange = intermediate risk ; G = green = no additional risk

Vitalityscore <= 3.27

| Gender = Male

| | Systolic bloodpressure = R: Yes, improve nutritional behavior (8.3)

| | Systolic bloodpressure = O

| | | Years working in function? <= 14: No, no advice to improve nutritional behavior (8.25/2.25)

| | | Years working in function? > 14: Yes, improve nutritional behavior (2.12)

| | Systolic bloodpressure = G

| | | Flexibility back = R

| | | | nutrition = R: Yes, improve nutritional behavior (3.3)

| | | | nutrition = O

| | | | | age <= 49.34: No, no advice to improve nutritional behavior (3.0)

| | | | | age > 49.34: Yes, improve nutritional behavior (2.0)

| | | | nutrition = G: Yes, improve nutritional behavior (0.0)

| | | Flexibility back = O

| | | | Physical endurance = R: Yes, improve nutritional behavior (37.69)

| | | | Physical endurance = O: No, no advice to improve nutritional behavior (3.23/0.23)

| | | | Physical endurance = G: Yes, improve nutritional behavior (15.08/1.0)

| | | Flexibility back = G: No, no advice to improve nutritional behavior (1.04/0.04)

| Gender = Female

| | smoking = R: No, no advice to improve nutritional behavior (1.0)

| | smoking = O: Yes, improve nutritional behavior (2.0)

| | smoking = G

| | | Years working in function? <= 3.76: Yes, improve nutritional behavior (2.0)

| | | Years working in function? > 3.76: No, no advice to improve nutritional behavior (5.0)

Vitalityscore > 3.27

| Vitalityscore <= 4.91

| | Contract = Fixed

| | | Physical endurance = R

| | | | Physical strain = R: Yes, improve nutritional behavior (7.45/1.0)

| | | | Physical strain = O

| | | | | BMI = R: Yes, improve nutritional behavior (2.44/0.4)

| | | | | BMI = O

| | | | | | cholesterol = R: Yes, improve nutritional behavior (1.02)

| | | | | | cholesterol = O: No, no advice to improve nutritional behavior (2.39/0.2)

| | | | | | cholesterol = G: Yes, improve nutritional behavior (10.6/1.4)

| | | | | BMI = G: No, no advice to improve nutritional behavior (4.08/0.08)

| | | | Physical strain = G

| | | | | Waist circumference = R: No, no advice to improve nutritional behavior (6.25/0.68)

| | | | | Waist circumference = O: Yes, improve nutritional behavior (12.97/2.98)

| | | | | Waist circumference = G: No, no advice to improve nutritional behavior (6.7/1.1)

| | | Physical endurance = O: No, no advice to improve nutritional behavior (17.15/3.88)

| | | Physical endurance = G: No, no advice to improve nutritional behavior (64.94/20.34)

| | Contract = Temporary contract: No, no advice to improve nutritional behavior (8.0)

| Vitalityscore > 4.91: No, no advice to improve nutritional behavior (64.0/3.0)

Number of Leaves (profiles) : 28

Size of the tree: 45
